# Supplementary material for: Optimization of Thymidine Kinase-Based Safety Switch for Neural Cell Therapy
Source: Cells. 2022 Jan 31;11(3):502. doi: 10.3390/cells11030502 (PMC8834506; doi:10.3390/cells11030502)
Supplement: Supplementary file 1 [file cells-11-00502-s001.zip › cells-1520019-supplementary.pdf]

Supplementary data:

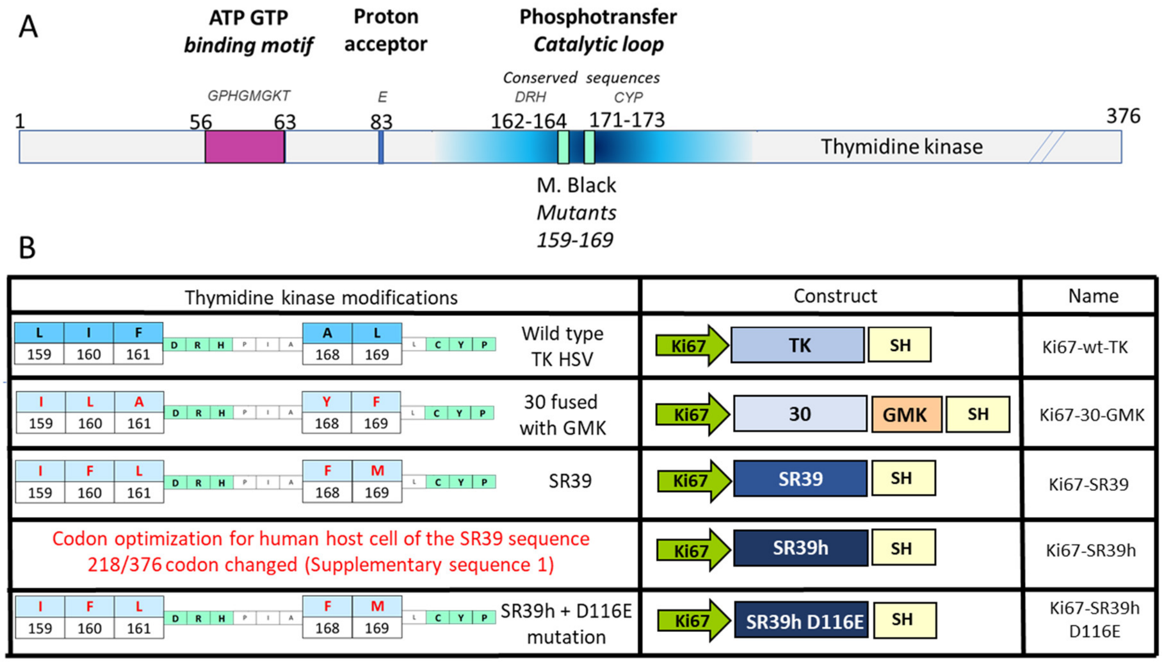

Supplementary Figure S1: Herpes simplex thymidine kinase (TK) variants used in this study.

A) Schematic representation of wild type TK; the two variants generated by Margaret Black (variant 30 and variant SR39) have amino changes close to the highly conserved region (162-164 and 171-173). B) Schematic representation of the different variants. Wild type TK is shown in the top lane, the two Margaret Black variants in lane 2 and 3, and two novel variants created for this study (SR39h and SR39h D116E) are shown in lane 4 and 5. Note that variant 30 was a fusion protein of a TK variant and guanylate kinase (GMK). The humanized SR39h variant codes for the same amino acid sequence as SR39. All variants had an N-terminal “she ble” protein (SH) extension conferring zeocin resistance to cell lines expressing the construct. For most experiments shown in our study, TK variants were expressed under the control of the Ki67 cell-cycle-dependent promoter.

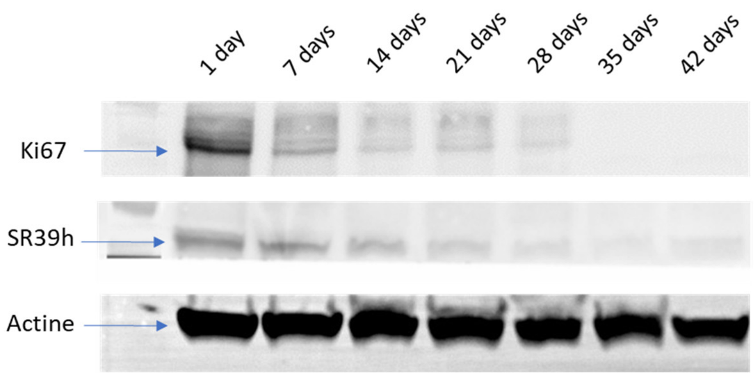

Supplementary Figure S2: Decreased of SR39h and Ki67 protein level during cell differentiation toward neurons. hESC expressing SR39h under the control of the Ki67 promoter were differentiated toward neurons during 42 days. Protein expression of SR39h and the endogenous Ki67 was analyzed by Western blot. The lower panel is a loading control with an anti-actin antibody.

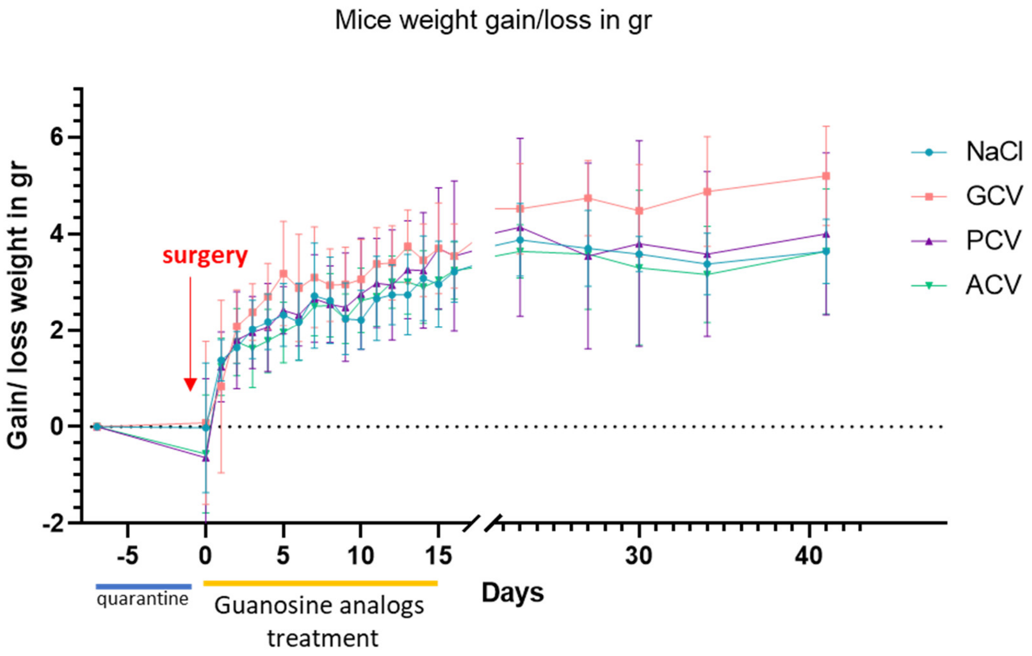

Supplementary Figure S3: Mice weight

Mice were weighed daily during guanosine analogs treatment to identify differences in weight gain/loss between treated groups. No differences were observed between treatment groups.

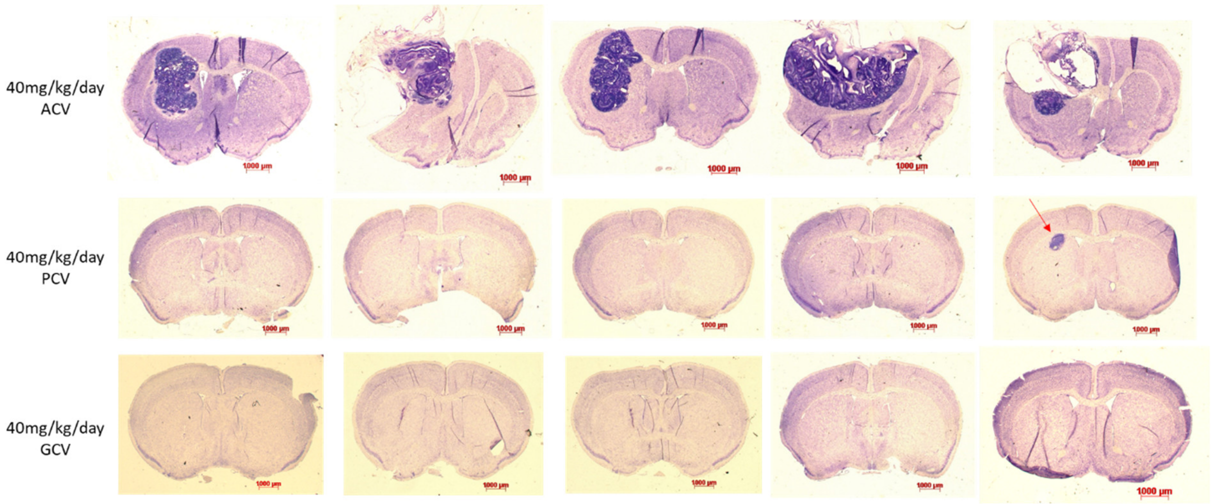

Supplementary Figure S4: *in vivo* validation of suicide gene. Coronal section of NOD/SCID mice 47 days after injection of hESC expressing the SR39h thymidine kinase variant. Mice were treated for 2x5 days with the respective nucleoside analogs (see Methods). Upper lane: ACV; middle lane: PCV; lower lane: GCV. Sections were colored with cresyl violet and dark purple staining reflects a high density of nuclei. Scale bar 1000 µm.

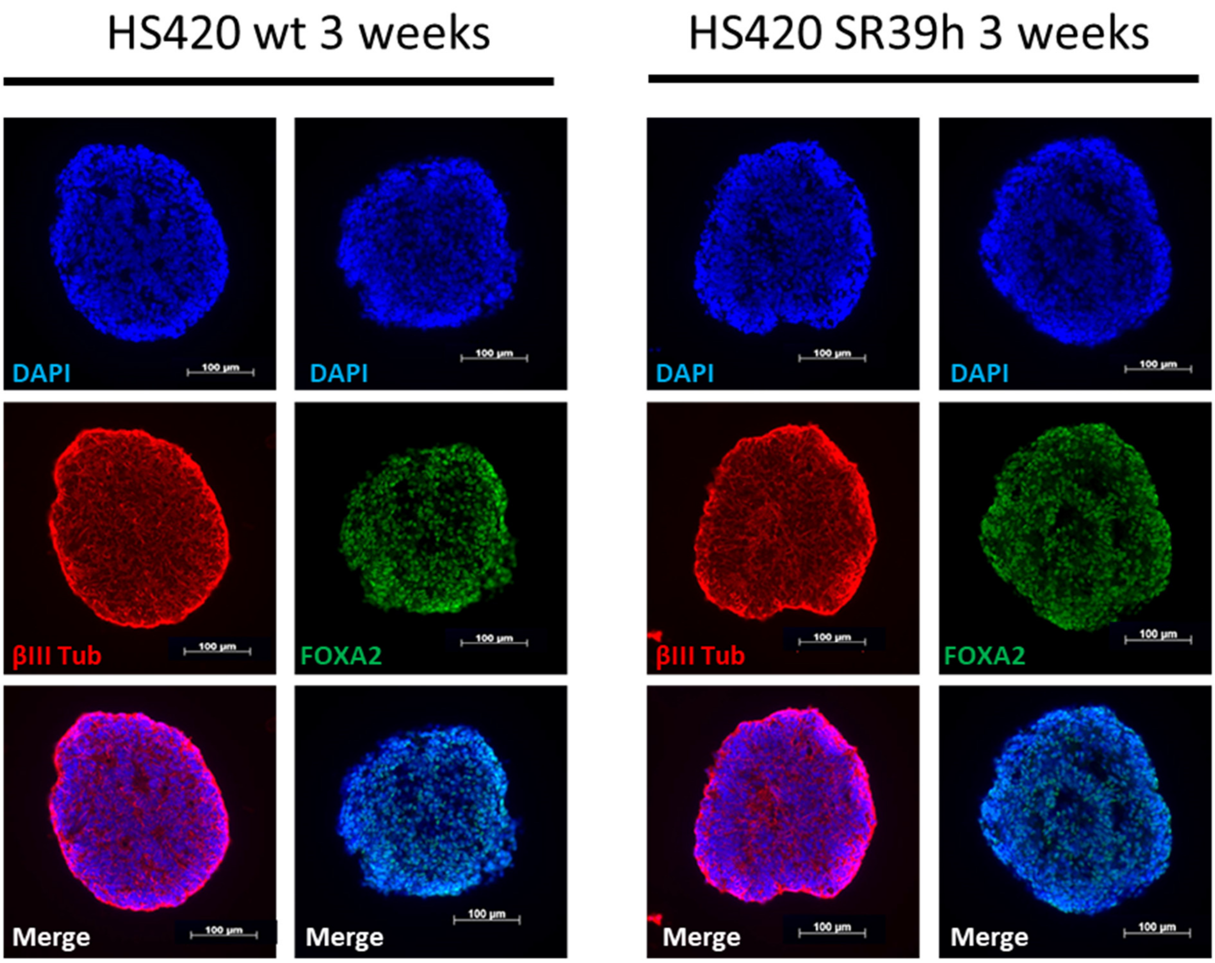

Supplementary Figure S5: ***hESC differentiation toward neurons.***

Wild type and SR39h expressing hESC were differentiated towards neurons during 3 weeks. Immunostaining: FOXA2 (mouse, Santa Cruz, sc-374376), beta-III tubulin (βIII Tub, mouse, Sigma, T8660), nuclei were counterstained with DAPI.

Supplementary sequence 1: **SR39h and SR39 sequence alignment.** Viral-TK DNA sequence was humanized using web-based algorithms (www.genscript.com), nucleotides changed in SR39h sequence were labeled in red.

```
>>SR39 1131 bp                                          (1131 nt)
 Waterman-Eggert score: 3359;  221.0 bits; E(1) <  3.7e-61
77.5% identity (77.5% similar) in 1129 nt overlap (1-1129:1-1129)

                10        20        30        40        50        60
SR39h   ATGGCCTCTTATCCTGGACACCAGCACGCCAGCGCCTTTGATCAGGCTGCCAGATCTAGA
        ::::: :: :: :: :: :: :: :::::    :: :: :: ::::::::  : ::: : 
SR39    ATGGCTTCGTACCCCGGCCATCAACACGCGTCTGCGTTCGACCAGGCTGCGCGTTCTCGC
                10        20        30        40        50        60

                70        80        90       100       110       120
SR39h   GGCCACAGCAACAGAAGAACAGCCCTGCGGCCTCGGAGACAGCAAGAGGCTACAGAAGTT
        ::::: :::::: :: : :: ::  :::: :::::  : :::::::: :: :: ::::: 
SR39    GGCCATAGCAACCGACGTACGGCGTTGCGCCCTCGCCGGCAGCAAGAAGCCACGGAAGTC
                70        80        90       100       110       120
```

```
                 130       140       150       160       170       180
SR39h     CGGCCCGAGCAGAAGATGCCCACACTGCTGAGAGTGTACATCGACGGCCCTCACGGCATG
          :: :: :::::::: :::::::: :: ::: : :: :: :: ::::: :: ::::: :::
SR39      CGCCCGGAGCAGAAAATGCCCACGCTACTGCGGGTTTATATAGACGGTCCCCACGGGATG
                 130       140       150       160       170       180

                 190       200       210       220       230       240
SR39h     GGCAAGACCACAACAACACAGCTGCTGGTGGCCCTGGGCAGCAGAGATGATATCGTGTAC
          :: :: ::::: :: :: :: :::::::::::::::::     : :: :::::::: :::
SR39      GGGAAAACCACCACCACGCAACTGCTGGTGGCCCTGGGTTCGCGCGACGATATCGTCTAC
                 190       200       210       220       230       240

                 250       260       270       280       290       300
SR39h     GTGCCCGAGCCTATGACCTATTGGAGAGTGCTGGGCGCCAGCGAGACAATCGCCAACATC
          :: :::::::: ::::: :: ::: : :::::::: ::   :::::::::::: ::::::
SR39      GTACCCGAGCCGATGACTTACTGGCGGGTGCTGGGGGCTTCCGAGACAATCGCGAACATC
                 250       260       270       280       290       300

                 310       320       330       340       350       360
SR39h     TACACCACACAGCACCGGCTGGATCAGGGCGAAATTTCTGCTGGCGACGCCGCCGTGGTT
          ::::::::::: ::::: :: :: ::::: :: :: :: :: :: ::::: :: ::::: 
SR39      TACACCACACAACACCGCCTCGACCAGGGTGAGATATCGGCCGGGGACGCGGCGGTGGTA
                 310       320       330       340       350       360

                 370       380       390       400       410       420
SR39h     ATGACATCTGCCCAGATCACCATGGGCATGCCTTACGCCGTGACAGATGCTGTGCTGGCC
          ::::::   :::::::: :: :::::::::::::: :::::::: :: :: :: ::::: 
SR39      ATGACAAGCGCCCAGATAACAATGGGCATGCCTTATGCCGTGACCGACGCCGTTCTGGCT
                 370       380       390       400       410       420

                 430       440       450       460       470       480
SR39h     CCTCACATTGGCGGAGAAGCCGGATCTTCTCATGCCCCACCTCCAGCTCTGACCATCTTC
          ::::: :: :: :: :: :: ::    :: :::::::: :: :: :: :: :::::::::
SR39      CCTCATATCGGGGGGGAGGCTGGGAGCTCACATGCCCCGCCCCCGGCCCTCACCATCTTC
                 430       440       450       460       470       480

                 490       500       510       520       530       540

SR39h     CTGGACAGACACCCTATCGCCTTCATGCTGTGTTACCCTGCCGCCAGATACCTGATGGGC
          :: ::: : :: :: ::::::::::::::::: ::::: :::::  : ::::: ::::::
SR39      CTCGACCGCCATCCCATCGCCTTCATGCTGTGCTACCCGGCCGCGCGGTACCTTATGGGC
                 490       500       510       520       530       540

                 550       560       570       580       590       600

SR39h     AGCATGACACCTCAGGCCGTGCTGGCTTTCGTGGCCCTGATTCCTCCTACACTGCCCGGC
          :::::::: :: :::::::::::::: ::::::::::: :: :: :: ::  ::::::::
SR39      AGCATGACCCCCCAGGCCGTGCTGGCGTTCGTGGCCCTCATCCCGCCGACCTTGCCCGGC
                 550       560       570       580       590       600

                 610       620       630       640       650       660

SR39h     ACCAATATCGTGCTGGGAGCCCTGCCTGAGGACCGGCACATTGATAGACTGGCCAAGAGA
          ::::: :::::::: :: ::::: :: :::::: : ::::: ::  : ::::::::  : 
SR39      ACCAACATCGTGCTTGGGGCCCTTCCGGAGGACAGACACATCGACCGCCTGGCCAAACGC
                 610       620       630       640       650       660

                 670       680       690       700       710       720
SR39h     CAGCGGCCTGGCGAGAGACTGGATCTGGCTATGCTGGCCGCCATCAGAAGAGTGTACGGC
```

```
        ::::: :: :::::: : ::::: :::::::::::::: :: ::  :  : :: :::::
SR39    CAGCGCCCCGGCGAGCGGCTGGACCTGGCTATGCTGGCTGCGATTCGCCGCGTTTACGGG
               670       680       690       700       710       720

               730       740       750       760       770       780
SR39h   CTGCTGGCCAACACCGTGCGGTATCTTCAATGTGGCGGCTCTTGGAGAGAGGACTGGGGA
        :: :: ::::: :: ::::::::::: :: :: ::::: :: ::: : ::::::::::::
SR39    CTACTTGCCAATACGGTGCGGTATCTGCAGTGCGGCGGGTCGTGGCGGGAGGACTGGGGA
               730       740       750       760       770       780

               790       800       810       820       830       840
SR39h   CAGCTTTCTGGCACAGCAGTTCCTCCACAAGGCGCCGAGCCTCAGTCTAATGCTGGACCC
        :::::::: :: :: :: :: :: :: :: :: :::::::: :::   :: :: :: ::
SR39    CAGCTTTCGGGGACGGCCGTGCCGCCCCAGGGTGCCGAGCCCCAGAGCAACGCGGGCCCA
               790       800       810       820       830       840

               850       860       870       880       890       900
SR39h   AGACCTCACATCGGCGACACCCTGTTTACCCTGTTCAGAGCCCCTGAGCTGCTGGCTCCT
         :::: :: ::::: :::::  : :::::::::::  : ::::: ::: ::::::: ::
SR39    CGACCCCATATCGGGGACACGTTATTTACCCTGTTTCGGGCCCCCGAGTTGCTGGCCCCC
               850       860       870       880       890       900

               910       920       930       940       950       960
SR39h   AACGGCGACCTGTACAACGTGTTCGCCTGGGCTCTTGACGTGCTGGCAAAGCGGCTGAGA
        :::::::::::::: :::::::: ::::::::  : :::::  :::: :: :: ::  :
SR39    AACGGCGACCTGTATAACGTGTTTGCCTGGGCCTTGGACGTCTTGGCCAAACGCCTCCGT
               910       920       930       940       950       960

               970       980       990      1000      1010      1020
SR39h   TCCATGCACGTGTTCATCCTGGACTACGATCAGTCCCCTGCCGGCTGTAGAGATGCTCTG
        ::::::::::: :: :::::::: ::::: :: :: :: ::::::::  : :: :: :::
SR39    TCCATGCACGTCTTTATCCTGGATTACGACCAATCGCCCGCCGGCTGCCGGGACGCCCTG
               970       980       990      1000      1010      1020

              1030      1040      1050      1060      1070      1080
SR39h   CTGCAGCTGACAAGCGGCATGGTGCAGACCCACGTTACAACCCCTGGCAGCATCCCCACC
        ::::: :: ::   ::: ::::: ::::::::::: :: ::::: :::  ::: :: ::
SR39    CTGCAACTTACCTCCGGGATGGTCCAGACCCACGTCACCACCCCCGGCTCCATACCGACG
              1030      1040      1050      1060      1070      1080


              1090      1100      1110      1120
SR39h   ATCTGTGACCTGGCCAGAACCTTCGCCAGAGAGATGGGCGAAGCCAACT
        :: :: ::::::::  : :: :: ::: : :::::::: :: :: ::::
SR39    ATATGCGACCTGGCGCGCACGTTTGCCCGGGAGATGGGGGAGGCTAACT
              1090      1100      1110      1120
```
